# Clonality, recombination, and hybridization in the plumbing-inhabiting human pathogen *Fusarium keratoplasticum* inferred from multilocus sequence typing

**DOI:** 10.1186/1471-2148-14-91

**Published:** 2014-04-26

**Authors:** Dylan PG Short, Kerry O’Donnell, David M Geiser

**Affiliations:** 1 Department of Plant Pathology, University of California, Davis, 1636 E Alisal St., Salinas, CA 93905, USA; 2 Bacterial Foodborne Pathogens and Mycology Research Unit, National Center for Agricultural Utilization Research, US Department of Agriculture, Agricultural Research Service, 1815 North University Street, Peoria, IL 61604, USA; 3 Department of Plant Pathology & Environmental Microbiology, The Pennsylvania State University, University Park, PA 16802, USA

**Keywords:** Biofilm, Clonality, *Fusarium*, Hybridization, MLST, Mycotic pathogen, Population dynamics, Recombination

## Abstract

**Background:**

Recent work has shown that *Fusarium* species and genotypes most commonly associated with human infections, particularly of the cornea (mycotic keratitis), are the same as those most commonly isolated from plumbing systems. The species most dominant in plumbing biofilms is *Fusarium keratoplasticum*, a cosmopolitan fungus known almost exclusively from animal infections and biofilms. To better understand its diversity and population dynamics, we developed and utilized a nine-locus sequence-based typing system to make inferences about clonality, recombination, population structure, species boundaries and hybridization.

**Results:**

High levels of genetic diversity and evidence for recombination and clonality were detected among 75 clinical and 156 environmental isolates *of Fusarium keratoplasticum*. The multilocus sequence typing system (MLST) resolved 111 unique nine-locus sequence types (STs). The single locus bifactorial determinants of mating compatibility (mating types *MAT1-1* and *MAT1-2*), were found in a ratio of 70:30. All but one of the 49 isolates of the most common ST (FSSC 2d-2) came from human infections, mostly of the cornea, and from biofilms associated with contact lenses and plumbing surfaces. Significant levels of phylogenetic incongruence were found among loci. Putative clonal relationships among genotypes were estimated, showing a mixture of large clonal complexes and unrelated singletons. Discordance between the nuclear ribosomal rRNA and other gene genealogies is consistent with introgression of ribosomal RNA alleles of phylogenetic species FSSC 9 into *F. keratoplasticum*. No significant population subdivision based on clinical versus non-clinical sources was found.

**Conclusions:**

Incongruent phylogenetic trees and the presence of both mating types within otherwise identical STs were observed, providing evidence for sexuality in *F. keratoplasticum*. Cryptic speciation suggested in a published three-locus MLST system was not supported with the addition of new loci, but evidence of introgression of ribosomal RNA genes from another strongly supported phylogenetic species (FSSC 9), also known from plumbing systems and human infections, was detected in two isolates. Overall, *F. keratoplasticum* is a diverse and geographically unstructured species with a mixed clonal and recombinant life history.

## Background

*Fusarium* is a large cosmopolitan genus of filamentous fungi best-known for causing a plethora of economically important plant diseases and mycotoxicoses [1]. Fusaria also cause human infections ranging from sinusitis, pneumonia, and localized skin lesions to life-threatening disseminated mycoses in immune-compromised and immune-suppressed individuals [2]. Along with *Aspergillus*, it is a frequent cause of trauma-associated mycotic keratitis in the tropics [3] and it is a predominant cause of contact-lens associated mycotic keratitis worldwide [4-9]. *Fusarium* is noted for its resistance to the broad-spectrum of antifungal drugs currently available [10]. Multilocus sequence typing (MLST) is a common and powerful tool for elucidating the diversity and population biology of microbial pathogens [11-13]. Several MLST systems have been developed for studying the genetic diversity and population biology of fungal human pathogens including, among others, *Aspergillus *[14], *Blastomyces *[15], *Candida *[16], *Coccidioides *[17], *Cryptococcus *[18], *Histoplasma *[19], and *Pneumocystis *[20]. Several studies utilizing MLST and genealogical concordance phylogenetic species recognition in *Fusarium* have revealed strongly supported species boundaries and have elucidated the spectrum of fusaria associated with human pathogenicity [4,9,12,21-27]. The application of MLST to mycotic pathogens has identified epidemic clones [28], uncovered signatures of linkage equilibrium associated with sexual recombination [28-30], and revealed cryptic speciation [15,31,32]. Typing systems are also valuable for epidemiological inference and for providing a framework for understanding the distribution of important phenotypes within species such as antimicrobial drug resistance [24].

Knowledge of species boundaries is a prerequisite for studying intraspecific population dynamics [33], and through several molecular systematics studies, the phylogenetic diversity of clinical fusaria has been elucidated [22-24]. Molecular markers useful for phylogenetics tend to utilize coding and intron sequences of various genes and have proved useful for distinguishing not only species, but also common intraspecific sequence types (STs) [26,34-36]. A three-locus MLST system developed for clinically relevant members of the species-rich *Fusarium solani* species complex (FSSC), which are all nested with Clade 3, utilizes an intron-rich portion of the translation elongation factor 1-alpha gene (TEF), a portion of the nuclear ribosomal RNA gene repeat that includes the internal transcribed spacer (ITS) regions and D1-D2 region of the nuclear large ribosomal RNA subunit gene (rDNA), and the gene encoding RNA polymerase II second largest subunit (*RPB2*) [24]. This system resolves ~60 phylogenetic species within the FSSC and distinguishes over 300 genetically distinct STs within Clade 3 of the FSSC [24].

*Fusarium* continues to be a nosocomial problem and MLST studies have highlighted the prevalence of certain widespread STs in hospital environments [37]. Members of the *F. oxysporum* species complex (*e.g.,* FOSC ST 33) [25,37] and the FSSC [38], specifically *F. petroliphilum* (*e.g.*, FSSC 1-a) and *F. keratoplasticum* (*e.g.*, FSSC 2-d) [39], are the fusaria most commonly identified in hospital water systems employing MLST. Clinically important STs within *F. keratoplasticum* and other fusaria appear to be ubiquitous in indoor plumbing [40,41]. Moreover, multiple STs that are associated with cases of contact lens-associated mycotic keratitis [4] may also be found in this environment [4,42]. In fact, based on current analyses [39,41], *F. keratoplasticum* is the single most common *Fusarium* species associated with human infections.

Although its monophyly is well-supported [9,21,24,43], *F. keratoplasticum* (FSSC 2) does not possess any distinctive morphological characters and is highly variable in culture [39], so morphological identification is impossible. Therefore, a three-locus MLST system was developed, which resolved 55 unique STs within *F. keratoplasticum* primarily from clinical sources [24]. In a subsequent expanded analysis, nearly half (107/231) of the isolates from clinical and environmental sources belonged to a single ST, FSSC 2-d [39]. In addition to supporting its monophyly, the combined three-locus phylogeny showed a moderately supported partition within *F. keratoplasticum*, suggesting that further genetic subdivision may exist within this species [24]. Also, evidence suggesting introgressive hybridization between *F. keratoplasticum* and a second FSSC species (FSSC 9) was reported [39].

To better understand the population biology of *F. keratoplasticum*, the three-locus MLST system was expanded to include six novel sequence-based markers, developed using the complete genome sequence of a related species, FSSC 11 (*F. solani* f. sp *pisi*) [44]. In addition, a PCR assay for mating-type determination [39] was utilized as a tenth locus to discriminate STs and investigate the potential for sexual reproduction. These markers were used to screen environmental and clinical isolates to infer the intraspecific diversity and relative roles of recombination, clonality, and hybridization in *F. keratoplasticum*.

## Methods

### Isolates of *F. keratoplasticum*

We analyzed 231 *F. keratoplasticum* isolates collected from a variety of sources and geographic origins in this study. Sources included human infections (N = 76), plumbing drains (N = 110), as well as veterinary infections, soil and various anthropogenic substrates (Additional file 1: Table S1). The human isolates presented in this paper have come from many sources over decades and were collected in a clinical setting. The isolates were sent from clinics for identification. We do not request or maintain personal information about the patients, and only keep data on the body part/disease associated with the isolate, and its general geographic source. Although isolations from six continents are represented, 84% of the isolates were from North America.

### Locus development and primer design

Six new sequence markers (loci 3968, 3972, 4081, 6512, 5437, and 6549) were developed in this study (Tables 1 and 2), which were utilized in combination with the existing 3-locus system [21,24,45]. Based on the optical map of *F. solani* f. sp. *pisi* (FSSC 11; also known as ‘*Nectria’ haematococca* Mating Population VI (NhMPVI)), the six new markers are predicted to reside on four different chromosomes. Markers were chosen based on the presence of single-nucleotide polymorphisms (SNPs) as well as microsatellites and insertion/deletion polymorphisms (indels) in 20 isolates of *F. keratoplasticum* and other species in the FSSC (Table 1). The complete genome was downloaded [46] and searched for 500–800 bp regions containing perfect microsatellite repeats using Tandem Repeats Finder [47]. Flanking primers were designed using Primer3 [48].

**Table 1:** Characteristics of loci employed

**Locus**^**1**^	**Genome position in MPVI**^**2**^	**P. I. chars.**^**3**^	**SNPs**^**4**^	**No. of indels/Msats: no. of alleles for each**^**5**^	**C. I.**^**6**^
*TEF*	sca_2_chr3_3_0: 2429595-2430256	18	23	4 : 3,3,2,2^s^	0.75
rDNA	Unknown	9	6*	None observed	0.9
*RPB2*	sca_20_chr6_4_0: 1850977-1852794	14	21	None observed	1
3968	sca_8_chr1_1_0: 1137337-1138068	11	17	2 : 2^s^, 2	0.95
3972	sca_82_chr10_2_0: 791230-792036	10	17	None observed	1
4081	sca_8_chr1_1_0: 544046-544635	13	28	5 : 2,5,2^s^,2^s^,2^s^	0.95
6512	sca_26_chr2_2_0: 74409-74990	9	12	2 : 2^s,^ 2^s^	0.93
5439	sca_37_chr_6_2_0: 374081-374828	6	8	4 : 2^s^,2,2^s^,2^s^	1
5437	sca_37_chr_6_2_0: 78042-78546	25	27	2 : 11,2	0.95
*MAT*	sca_2_chr3_3_0: N.A.	N.A.	N.A.	N.A.	N.A.

1. Name of locus 2. Scaffold, chromosome and inclusive base pairs of targeted sequences in *Fusarium solani* f. sp. *pisi*. 3. Number of parsimony informative characters observed within *F. keratoplasticum.* 4. Number of single nucleotide polymorphisms observed within *F. keratoplasticum.* 5. Number of total insertion/deletion and microsatellite polymorphisms observed within *F. keratoplasticum* (underlined), followed by the number of alleles observed at each, i.e. locus 5437 contains 2 such polymorphisms, one of which has 11 alleles. Superscript s indicates that this polymorphism was found in only one isolate. 6. Consistency index of individual locus maximum parsimony trees. *Putative hybrids contained 4 more SNPs within their rDNA sequences.

**Table 2:** Primer pairs used for PCR amplification and Sanger sequencing

**Locus**	**Fw primer**	**Rv primer**	**Annealing temp. (°C)**	**Size**^**1 **^**(bp)**	**Seq. composition (bp)**^**2**^	**Alleles**^**3**^	***H***^**4**^
*TEF*	EF1: ATGGGTAAGGARGACAAGAC	EF2: GGARGTACCAGTSATCATGTT	53	667	256 intronic; 411 exonic	22	0.89
rDNA	ITS5: GGAAGTAAAAGTCGTAACAAGG	NL4: GGTCCGTGTTTCAAGACGG	53	1029	705 coding; 324 ITS	6	0.35
*RPB2* 5-7	5f2: GGGGWGAYCAGAAGAAGGC	7cr: CCCATRGCTTGYTTRCCCAT	55	863	Exonic	11	0.51
*RPB2* 7-11	7cf: ATGGGYAARCAAGCYATGGG	11ar: GCRTGGATCTTRTCRTCSACC	55	881	Exonic	-	-
3968	3968fw: TGTTGGTTCGAGATGGTTGA	3968rv: GAGAAGGGCAACTGGGAGA	53	770	Intergenic	12	0.68
3972	3972fw: TCTGATGCAGACTAGCACTCG	3972rv: ATCGGACGAAACAGAGCAGG	53	831	Intergenic	13	0.45
4081	4081fw: TGACRAGGATGAATGAGCGA	4081rv: TGACCAGCCTCCAAGSG	56	642	Intergenic	18	0.8
6512	6512fw: GGAGGACCAGGAGGAATAGG	6512rv: CAAAGCAGATCGACTGAGGA	53	644	Intergenic	12	0.52
5439	5439fw: AATGGGAATACGAGCGTCAG	5439rv: AGGGGCTGCTGTTAGTGAGA	53	779	Intergenic	10	0.48
5437	5437fw: AACAAGACAAGGCAGCAGGT	5437rv: TCCAGAGGAACGACGAGGC	56	544	213 exonic, 331 intergenic	23	0.88
*MAT1-1*	MAT1-S-1 F: ATGGCTTTCCGCAGTAAGGA	MAT1-S-1R: CATGATAGGGCAGCAAAGAG	53	~200	N. A.	N. A.	N. A.
*MAT1-2*	MAT2-S-1 F: GGGAATCTGAGAAAGATACGTAC	MAT2-S-1R: CGGTACTGGTAGTCGGGAT	53	~800	N. A.	N. A.	N. A.

1. Number of characters in the aligned dataset. For different isolates, the exact length of any region may be variable due to indels and microsatellite repeats 2. ITS = internal transcribed spacers 1 and 2. Locus 5437 contains coding regions of a predicted hypothetical protein of unknown function. 3. Number of alleles observed in *F. keratoplasticum* 4. Average genetic diversity of each of the 9 loci calculated using LIAN accessed at http://pubmlst.org.

### Nucleic acid manipulation

Isolates were grown in 5 mL of potato dextrose broth (PDB) in test tubes for 14 days. Mycelium was then collected, rinsed with sterile distilled water, dried with paper towels, and transferred to 2 mL tubes for lyophilization. Mycelium was ground to a fine powder using plastic pestles and total genomic DNA was isolated using a DNeasy Plant Minikit protocol (Qiagen, Valencia, CA, USA) and suspended in a 100 μl volume of elution buffer. PCR and sequencing was performed using GoTaq PCR Kits (Promega, Madison, WI, USA) in 50 μl volumes following the manufacturer’s instructions, with reactions subjected to 2 minutes at 94°C, 35 cycles of 1 minute at 94°C, 1 minute at the appropriate annealing temperature (Table 2), and 1 minute at 72°C, followed by 10 minutes at 72°C. PCR products were sequenced at the Penn State Nucleic Acid Facility, Huck Institute of Life Sciences, University Park, PA. A multiplex PCR for identifying MAT idiomorph was performed as previously described [39]. PCR products were visualized using a 1.5% w/v agarose gel and scored as *MAT1-1* or *MAT1-2* based on amplicon size (~200 and 800 bp, respectively). Portions of the nuclear ribosomal intergenic spacer region (IGS) were also amplified and sequenced in two putative hybrid isolates as previously described [22] using Invitrogen PlatinumTaq kits (Life Technologies, Carlsbad, CA USA) following the manufacturer’s instructions.

### Sequence type (ST) diversity

Isolates were assigned to existing and novel STs as described previously [4,9,24]. Briefly, ST assignments were based on their position in terminal nodes in one of 200 maximum parsimony (MP) phylogenetic trees generated by implementation of the parsimony ratchet [49] on concatenated DNA sequence alignments in PAUP*4.0b. Chromatograms were then cross-checked for accuracy to confirm ST membership. Missing or ambiguous DNA positions were assigned Ns in the sequence alignments and treated as missing data. For the nine-locus MLST system, the previously assigned three-locus STs were amended with numerals indicating additional subdivision revealed by the six additional loci. For example, FSSC 2-d, the most common ST in the three-locus system, was subdivided into 18 nine-locus STs designated FSSC 2-d1 through FSSC 2-d18. SNP and insertion-deletion polymorphisms in the loci were used to distinguish STs. For certain MLST analyses, alleles at each locus were assigned simple numerical identifiers, which we refer to as digitized nine-locus STs; *e.g.,* 16-1-1-4-6-2-3-4-13 represents a combination of unique alleles observed at the nine loci in ST FSSC 2-c1. 24/2079 (1.2%) of the nine loci sampled across all isolates produced partial or no sequence data. There were 21 and three isolates missing a significant portion of one or two loci, respectively, and 11 of these cases involved locus 5439. When determining the number of multilocus STs, none were scored as unique by virtue of missing or ambiguous nucleotide data. The number of nine-locus STs was calculated using the digitized nine-locus STs as input into the application DNAcollapser implemented in the website FaBox [50]. Mating type was then used as a tenth locus, with individuals assigned as 1 or 2 based on the detection of the *MAT1-1* or *MAT1-2* idiomorph and the number of ten-locus STs was calculated in the same way. Nine-locus ST diversity of the total sample was estimated using the formula [*n*/(*n* - 1)](1 - ∑*x*_*i*_^2^), where *x*_*i*_ is the frequency of the _*i*_th ST and *n* is the number of isolates [51]. Genetic diversity of the loci and mean genetic diversity were calculated from the digitized nine-locus STs using LIAN [52] accessed at (http://pubmlst.org). To test for patterns of population differentiation, the dataset of 231 isolates was divided into two groups comprising clinical (n = 76) and non-clinical (n = 155) isolates. These groups were clone corrected and an exact test of population differentiation was performed in Arlequin ver 3.11 [53] using the digitized nine-locus STs. Finally, a numerically coded, clone corrected nucleotide sequence dataset with constant characters removed was used for plotting the mean number of genotypes vs. the number of loci in MULTILOCUS, by randomly sampling 1–182 characters 100 times each [54]. Sequence data for the six new loci developed in this study have been deposited in NCBI GenBank (Table 3).

**Table 3:** **NCBI GenBank accessions for DNA sequence data for six loci developed for *F. keratoplasticum***

**Locus**	**GenBank accession numbers**
3968	JN585993-JN586184
3972	JN586185-JN586372
4081	JN586373-JN586556
5437	JN586557-JN586711
5439	JN586712-JN586899
6512	JN586900-JN587087

### Estimation of clonal relationships

To generate population snapshots and to estimate clonal relationships among genotypes, the set of 111 unique digitized nine-locus STs was used as input into an MLST analysis [55] implemented using the web application eBURST [56], at the single locus variant (SLV) level. The total set of 231 digitized nine-locus STs was also analyzed at the triple locus variant (TLV) level utilizing the goEBURST algorithm [57] implemented in PHYLOVIZ [58]. STs that were not part of any TLV group were added manually as standalone data points. Mating-type data and source information were manually superimposed upon the resulting display of the TLV analysis with high-level edges (connections between STs identical at 7/8 and 6/8 loci) displayed as dashed and dotted lines, respectively. STs of isolates previously reported as producing cyclosporins *in vitro *[39,59] were indicated on the population snapshots in order to visualize those associated with this phenotype.

### Multilocus tests for linkage disequilibrium

To test for the non-random association of alleles, a clone corrected, digitized nine-locus ST dataset was used to calculate the Index of Association (*I*_*A*_) [60] together with rBarD, an alternative measure of *I*_A_ adjusted for the number of loci, using MULTILOCUS ver. 1.3b [54]. The observed *I*_*A*_ and rBarD values were calculated for the nine-locus dataset and compared to 1000 randomized datasets. In addition, a standardized *I*_*A*_ was calculated using LIAN [52]. Additionally, to account for potential effects due to physical linkage, *I*_*A*_ and rBarD were recalculated on clone corrected datasets with each of the nine loci removed separately.

### Detection of recombination

A clone corrected, concatenated NEXUS data file with constant characters removed was used to generate a NeighborNet in SPLITSTREE4 [61], and to calculate the dataset’s δ score (a measure of treelikeness, where higher δ values may reflect higher levels of recombination) [62] and phylogenetic diversity, a measure of diversity based on branch lengths [63]. A clone corrected, concatenated dataset including all nucleotide characters was used to perform the PHI test for recombination [64] implemented in SPLITSTREE4. Incompatibility among all 9 individual gene trees was assessed using COMPAT.PY, a program to detect topological conflict between supported clades in phylogenetic trees [65]. For each of the nine loci, one of 200 MP trees generated using PAUPRAT [49] with branch lengths included was used as input in a test for topological conflict.

### Detection of interspecific introgression

Based on the discovery of two putative hybrids of *F. keratoplasticum* (FRC S-2406 and FRC S-2509) that possess identical rDNA sequences to those of FSSC 9 [39], sequence data from the nine-locus MLST system were generated for three members of FSSC 9 (FRC S-2485, FRC S-2530, FRC S-2540). For comparative purposes, portions of the nine loci were also sequenced for FSSC 5 (FRC S-2519), *F. petroliphilum* (FSSC 1, FRC S-2550), and *F. solani* f. sp *pisi* (FSSC 11) (http://genome.jgi.doe.gov/Necha2/Necha2.home.html). Individual unrooted MP gene trees were generated as described previously to assess whether other loci of the two putative hybrid isolates showed affiliation to FSSC 9. Finally, a portion of the intergenic spacer region of the nuclear ribosomal RNA gene repeat (IGS) was sequenced for the two putative hybrids, three FSSC 9 isolates, and one *F. keratoplasticum* isolate (NRRL 32710) and analyzed to determine whether this portion of the rRNA repeat was also introgressed.

## Results

### *F. keratoplasticum* shows high levels of ST diversity

**Figure 1:**
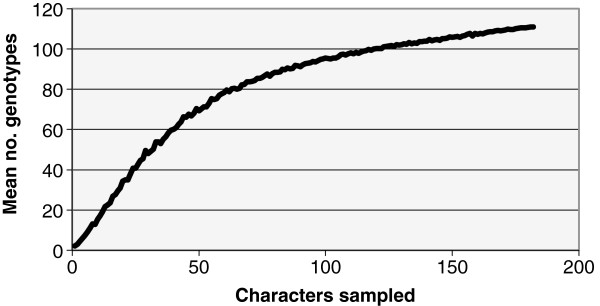
**Plot of mean number of genotypes of *F. keratoplasticum *** **as a function of number of characters (SNPs and indels) sampled.**

The nine-locus genetic diversity ranged from 0.35 to 0.89 with an average of 0.62 (Table 3). One hundred and eleven nine-locus and 122 ten-locus STs (with mating-type as a tenth marker) reflected high levels of diversity in the total sample of 231 isolates. The number of *MAT1-1* and *MAT1-2* isolates observed was 162 (70%) and 69 (30%), respectively. Isolates of seven STs comprised both mating types (2d1, 2d2, 2d3, 2f2, 2f6, 2 k4, 2o2). ST diversity was 0.94 among the nine-locus STs, and phylogenetic diversity (PD) of the nine-locus dataset calculated in SPLITSTREE4 was 0.45 (Table 3). Even with 9 loci covering ~7650 bp, the plot of genotypes vs. number of loci sampled indicates a likelihood of more diversity present within this panel of isolates than was resolved (Figure 1). No strong evidence for cryptic species boundaries within *F. keratoplasticum* was observed, as comparisons between gene trees for each of the nine loci did not reveal any groups with bootstrap values >60% in all of the MP trees (data not shown). No population differentiation was detected using exact tests comparing ST diversity of isolates from clinical sources with those from all other sources.

### Inferred relationships among clones and clonal complexes

**Figure 2:**
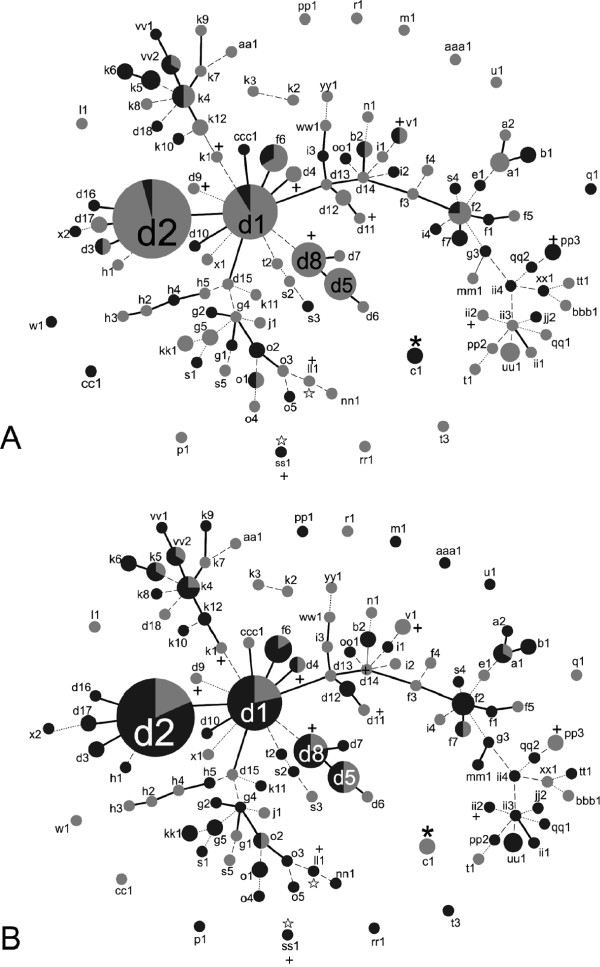
**Population snapshots of *F. keratoplasticum *inferred using Phyloviz beta.** Each node represents a unique ST (the prefix ″2-″ has been omitted for clarity); areas of nodes are proportional to the number of isolates. STs with 8/9 loci in common (SLVs) are connected with solid black lines, STs with 7/9 loci in common (DLVs) are connected with dashed lines, STs with 6/9 loci in common (TLVs) are connected with dotted lines. STs different at more than three loci not connected to a group. 9 SLV groups are shown. In **A)** light gray indicates proportion of STs that are *MAT1-1*; darker gray shading indicates proportion of STs that is *MAT1-2*. In **B)** light gray indicates isolates from clinical sources; darker gray indicates isolates from all other sources. Plus signs denote several STs containing isolates known to produce cyclosporin *in vitro*. Asterisks denote the ST of the ISO standard ATCC 36031 strain. White stars denote STs containing isolates successfully crossed in mating experiments.

Evolutionary relationships among the 231 *F. keratoplasticum* isolates inferred using goeBURST resolved nine distinct clonal complexes (i.e*.* groups of hypothetically clonally related genotypes or clonets). These comprised 50 STs that were defined at the single locus variant (SLV) level (STs identical at 8/9 loci, identified by solid black lines) (Figure 2). Double and triple locus variants (DLV and TLV; STs identical at 7/9 and 6/9 loci, respectively) were also identified by dashed and dotted lines, (Figure 2). Lastly, goeBURST resolved 14 STs comprising 15 isolates that were different at four of more loci (displayed as unconnected STs in Figure 2). Interestingly, included among the singleton STs were the isolate that is the International Organization for Standardization (ISO), ATCC 36031 (ST 2-c1), as well as one of only two confirmed sexually fertile isolates of *F. keratoplasticum* (ST 2-ss1) [39]. Isolates previously known to produce cyclosporins *in vitro* were found to belong to *F. keratoplasticum* STs 2-d4, 2-d8, 2-d9, 2-d11, 2-ii2, 2-ll1, 2-k1, 2-pp3, 2-ss1, and 2-v1 (Figure 2).

### Evidence for non-random association of alleles

Multiple analyses revealed a statistically significant association of alleles among loci, indicating that isolates may be related through asexual processes of population evolution. The index of association (*I*_*A*_) and rBarD calculated from the nine-locus dataset of digitized STs were 0.47 and 0.06, respectively; both differed significantly from the ranges of these values observed in 1000 artificially recombined datasets (p < 0.01) (Table 4). A standardized (*I*_*A*_) [52] of 0.059 was calculated using LIAN, and the null hypothesis of linkage equilibrium was rejected using 1000 resamplings in Monte Carlo and parametric tests (p < 0.001 and p < 3×10^-21^, respectively). To assess the potential contribution of linkage between loci, eight separate tests for linkage disequilibrium were also performed on clone corrected datasets with each locus removed individually. Each of these tests showed significant amounts of linkage disequilibrium (results not shown).

**Table 4:** **Summary statistics for the *F. keratoplasticum *data set**

No. isolates	231
No. nine-locus STs	111
*MAT1-1:MAT1-2* (total)	162:69
*MAT1-1*:*MAT1-2* (without 2-d types)	64:60
Allelic diversity^1^	0.94
Average genetic diversity of loci^2^	0.62
NeighborNet δ score^3^	0.28
Phylogenetic Diversity^4^	0.45
Index of Association^5^	0.47
rBarD^6^	0.06
Standardized *I*_*A*_^*7*^	0.05
No. of eBURST SLV groups (clonal complexes)	9
No. STs connected at SLV level	50
No. isolates in SLV level STs	162
No. singletons at SLV level	69

1. ST diversity calculated from Nei (43). 2. Average genetic diversity of the 9 loci calculated using LIAN (25). 3. Average δ score (26) of the clone corrected set of 111 unique STs calculated using SPLITSTREE. 4. Phylogenetic diversity (19) of the clone corrected set of 111 unique STs calculated using SPLITSTREE. 5. Index of Association (41) calculated using MULTILOCUS 1.3b (1) was found to be significantly different (p < 0.01) than 1000 randomizations, which showed an expected range of 2.86-4.09 (mean = 3.46). 6. rBarD calculated using MULTILOCUS 1.3b was found to be significantly different (p < 0.01) than 1000 randomizations, which showed an expected range of 0.0208-0.0283 (mean = 0.024). 7. Standardized *I*_*A*_ calculated using LIAN (25); the null hypothesis of linkage equilibrium was rejected using 1000 resamplings in Monte Carlo tests and a parametric test, with p < 0.001 and p < 3×10^21^ respectively.

### Evidence for recombination

**Figure 3:**
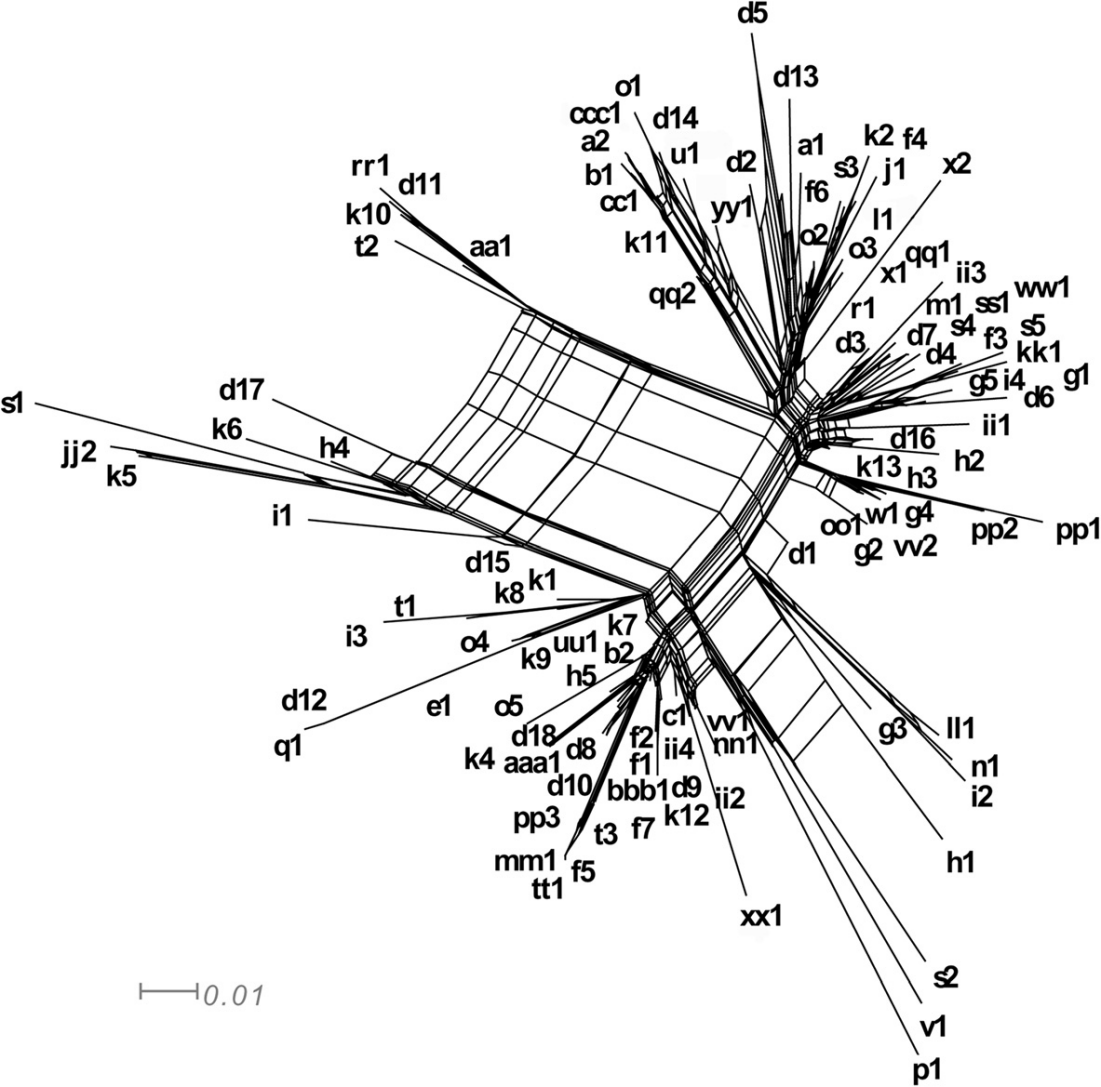
**Neighbor net of 111 unique nine-locus haplotypes inferred using SPLITSTREE4.** A PHI test for recombination (7) implemented in SPLITSTREE4 suggests a statistically high likelihood of recombination in the full dataset (p < 0.0001).

A net-like and reticulated NeighborNet was observed (Figure 3) and the PHI test for recombination implemented in SPLITSTREE4 supported the presence of recombination (p < 0.0001). The average δ score for the 111 unique STs using the concatenated dataset showed a significant departure in treelikeness from zero (δ = 0.28) and could be due to the presence of historical recombination events (26). Individual *F. keratoplasticum* gene trees indicated various levels of intralocus homoplasy, with consistency indices (CI) ranging between 0.75 in TEF to 1.0 in *RPB2*, 3972, and 5439 (Table 1). COMPAT.PY detected multiple topological conflicts among the trees inferred from the nine loci (results not shown).

### Introgression between phylogenetic species

**Figure 4:**
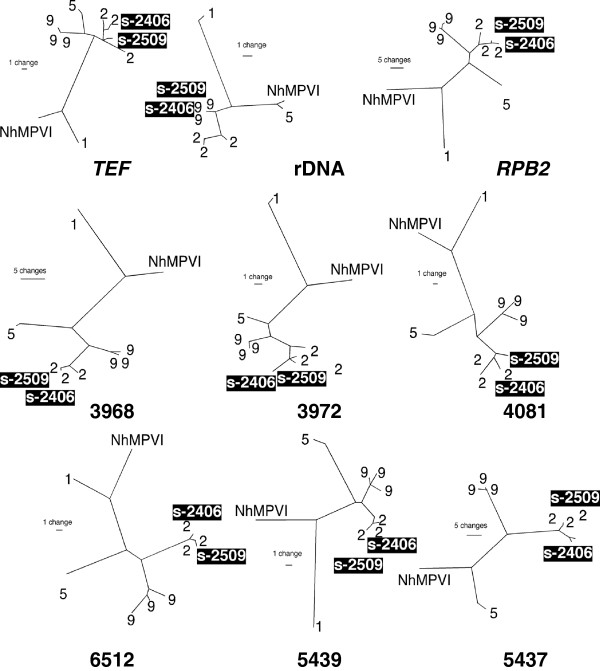
Nine individual locus unrooted maximum parsimony trees inferred using parsimony ratchet (72, 73) implemented in PAUP* 4.0. Isolates S-2406 and S-2510 are strongly supported members of *F. keratoplasticum* (FSSC 2) in eight of the trees*.* In the rDNA tree, however, these two isolates are strongly supported members of FSSC 9.

*F. keratoplasticum* isolates FRC S-2406 (from Honeymoon Island Park in Florida) and FRC S-2509 (from a shopping center in Georgia) possessed nuclear rDNA regions (ITS, the D1-D2 region of the large subunit, and IGS) that were perfect matches to those found in FSSC 9, suggesting some kind of genetic transfer between *F. keratoplasticum* and this species [39,41]. Phylogenetic analysis of the eight other loci grouped these isolates with *F. keratoplasticum* (Figure 4). Taken together, these analyses indicated that FRC S-2406 and FRC S-2509 possess ribosomal RNA repeats that are derived from FSSC 9, while the other eight loci have clear origins in *F. keratoplasticum*. Excluding the rDNA of these putative hybrids, the other loci supported reciprocal monophyly between *F. keratoplasticum* and FSSC 9.

## Discussion

The primary goal of this study was to apply novel sequence based markers to understand the diversity and population biology of *F. keratoplasticum*, the most common clinically relevant *Fusarium *[21,24]. In addition to their utility in addressing questions related to their basic ecology and epidemiology, MLST in *F. keratoplasticum* provides a framework for the study of biologically relevant phenotypes. For example, *F. keratoplasticum* STs vary in the types of secondary metabolites produced, including cyclosporins [39,59] (Figure 2). Sexual fertility also appears to be a variable phenotype within *F. keratoplasticum*. Perithecium formation was previously reported as very rare in crosses of opposite mating types, although a cross between STs 2-ss1 and 2-ll1 produced recombinant ascospores under laboratory conditions [39]. Finally, biofilm formation in *F. keratoplasticum* is another variable trait with practical significance. Although NRRL 22641 (=ATCC 36031) was recommended by the International Organization for Standardization guidelines for testing disinfectants, it does not form biofilms in *in vitro* models [66] and this singleton (ST 2-c1) appears to be quite divergent (Figure 2) and aberrant morphologically [39], casting further doubt on its choice for testing the efficacy of antimicrobials. It is unknown whether this isolate’s failure to form biofilms can be attributed to mutation, perhaps post-isolation, or phenotypes segregating in natural populations. In addition to those reported here, further tests of ATCC 36031 in comparison to other clinical isolates has led to the recommendation that multiple clinical isolates be utilized in disinfectant testing [67].

### High levels of sequence type diversity in *F. keratoplasticum*

Polymorphisms within the nine loci employed in this study ranged from 6–33 alleles per locus. The six new markers developed in this study were originally targeted because they harbored microsatellite repeats in the *F. solani* f. sp *pisi* genome. Sequencing these regions in *F. keratoplasticum* revealed high levels of nucleotide polymorphisms in addition to polymorphic repeats, motivating us to utilize them more fully as sequence-based markers and also to avoid issues of convergence that can be problematic in interpreting alleles based on variable numbers of tandem repeats [68]. SNPs were the most common polymorphisms observed within *F. keratoplasticum* (163 out of ~7650 characters). Polymorphism per region sequenced was higher than observed in MLST systems of other fungi pathogenic to humans such as *Aspergillus fumigatus *[14], but lower than the dimorphic mycotic pathogens *C. albicans, C. tropicalis and C. glabrata* (6). Testing the primers designed in this study against other FSSC species indicated utility of these markers broadly in the complex (Figure 4), as they successfully amplified DNA from *F. petroliphilum* (FSSC 1), *F. falciforme* (FSSC 3 + 4), and FSSC phylogenetic species 5, 9, 18, 22 and 24. Many polymorphisms were found in other FSSC species, especially in the microsatellite repeats, which were extremely variable between species, though the same repeats motifs were often monomorphic within *F. keratoplasticum.* Although the six new loci developed in this study consisted almost entirely of non-coding sequence residing in intergenic regions, the level of genetic diversity observed at these loci within *F. keratoplasticum* were surprisingly similar to that of the protein-coding genes (Table 2).

Previous MLST studies indicated that environmental and human pathogenic fusaria are diverse, spanning multiple species complexes, species, and STs within species, but certain STs have been found to be dominant. For example, *Fusarium oxysporum* species complex ST 33 was identified as one such widespread clonal lineage based on analysis of *TEF* and the IGS region. Moreover, analysis of amplified fragment length polymorphisms (AFLPs) revealed seven distinct haplotypes within ST 33 [25]. We observed a similar trend within *F. keratoplasticum* with the addition of six markers to the 3-locus MLST scheme. The majority of previously defined 3-locus STs were further resolved into multiple nine-locus STs (Table 1), some of which appeared to be quite distinct (Figures 2 and 3). Furthermore, the plot of genotypic diversity vs. number of loci sampled indicated that additional diversity in the nine-locus STs remains to be resolved (Figure 1).

### *F. keratoplasticum* has a mixed reproductive mode

Analyses of *F. keratoplasticum* isolates revealed evidence of clonality and recombination, mirroring reproductive modes observed in this species. FSSC species reproduce by clonally via asexual propagules (*e.g*., macroconidia, microconidia, chlamydospores) and several sexually through formation of perithecia and recombinant ascospores These potential modes of reproduction are known in *F. keratoplasticum *[39]*.* In contrast to the hypothesis of local sexual neighborhoods, where mating is thought to occur only between isolates of similar genotypes [69], isolates of *F. keratoplasticum* that produced recombinant ascospores shared only one identical sequence (locus 5439) among the nine loci. The observed frequencies of mating-types in *F. keratoplasticum* in the total sample deviated significantly from the expected 1:1 ratio (162:69 *MAT1-1*:*MAT1-2;* binomial cumulative probability p < 0.0001). This aberration may be due to sampling of the most common widespread clones, because when all 2-d types were removed, a ratio of 64:60 *MAT1-1*:*MAT1-2* (binomial cumulative probability p > 0.39) was observed.

A robust estimate of the contribution of mutation relative to recombination may be calculated as the ratio of STs that form complexes of SLVs, which are assumed to have arisen via mutation, to those that do not. In *F. keratoplasticum*, 50 STs resolved into 9 clonal complexes of SLVs, comprising 2–22 unique STs each (Figure 2). The remaining number of STs that did not resolve into SLV clonal complexes was 61, giving an inferred 1:1.24 ratio of mutation:recombination. Without clone correction, however, 162 isolates are connected at the SLV level compared to 69 isolates that are identical at fewer than 8/9 loci, giving an inferred mutation:recombination ratio of 2.3:1. This estimate contrasts with the situation observed in the more clonal *Talaromyces marneffei*, where the mutation:recombination ratio was approximately 5:1 [69,70]. A mixed clonal/recombinant structure has also been reported in clinically relevant fungi including *Aspergillus fumigatus*[32]*, Cryptococcus gattii *[29], *Candida albicans *[30,71], *Candida glabrata *[72], and *Paracoccidioides brasiliensis *[73].

### *F. keratoplasticum* ST 2-d as a transcontinental epidemic clone

As previously mentioned, *F. keratoplasticum* 2-d is the three-locus ST most commonly associated with plumbing fixture biofilms, including hospital water systems, and human infections. It comprised 46% of the isolates in our dataset (107 isolates), and despite the incomplete resolution from nine loci, it is retained as a group of related, common and geographically widespread STs. The nine-locus MLST system divided 2-d into 18 unique types, all but one of which are connected through a series of SLVs, DLVs and TLVs (Figures 3 and 2). Based on sequence information, the dominant subtypes 2-d1 and 2-d2 appear to represent a widespread clonal lineage found in the United States, Puerto Rico, Germany, and Qatar (Table 1). Additional evidence of a strong clonal component was provided by the discovery that 94% of 2-d1 and 2-d2 isolates were *MAT1-1* (Figure 2A). The remaining 6% of 2-d1 and 2-d2 isolates may represent one or multiple separate evolutionary lineages that have not been resolved, even with nine loci. The presence of haplotypes that are both extremely common and widely distributed has been observed in mycotic pathogens such as *Cryptococcus gattii *[28], in which the same common clone (*C. gattii* ST7) is present in Australia and the United States. In contrast, ST 2-k, previously shown to be among the six most common biofilm-associated human pathogenic fusaria [41], showed evidence for historical recombination (Figures 3 and 2), as 2-k2 and 2-k3 were members of an unconnected TLV group in the eBURST analysis (Figure 2). In support of this, 2-k isolates show a 9:10 *MAT1-1*:*MAT1-2* mating-type ratio, which is similar to the 1:1 ratio expected among randomly mating populations. However, interfertility of the 2-k isolates has not been tested.

### Introgression and hybridization

Introgression has been observed in other medically important fungi including *Candida* spp. [74], *Cryptococcus *[75], and *Coccidioides *[76], and is hypothesized to play an important role in producing diversity. Here, strong evidence of genetic exchange between two isolates of *F. keratoplasticum* and the unnamed species FSSC 9 is presented. FRC isolates S-2406 and S-2509 possessed *RPB2* and *TEF* alleles that clearly placed them in *F. keratoplasticum*, but their rDNA appeared to have been derived from a FSSC 9 parent (100% sequence identity to all known FSSC 9 haplotypes). *F. keratoplasticum* and FSSC 9 are closely related, and both occur in plumbing biofilms, so the simplest explanation for this is introgression via an interspecific hybrid cross. Analyses of these putative hybrids showed that they possessed typical *F. keratoplasticum* alleles at all other loci, suggesting that if the introgression of rDNA was due to a hybridization event, the isolates were likely heavily backcrossed into the *F. keratoplasticum* background. Hybridization between closely related, phylogenetically defined species has been inferred in *Fusarium* in multiple instances [77-80]. The introgressed FSSC 9 rDNA included the ITS regions, the large subunit rRNA, and IGS region, and may encompass the entire rDNA gene repeat. Genome scale analyses are needed to fully assess what portion of the hybrid genomes was derived from a FSSC 9 parent. These analyses may help elucidate whether this introgression was mediated by a sexual or parasexual process. It is also possible that other unorthodox mechanisms may be at play in generating genetic diversity in *F. keratoplasticum.* Species that possess small conditionally dispensible (CD) chromosomes are known to be present within the FSSC [81] as well as in other fusaria [77]. The genome of *F. solani* f. sp *pisi* includes three CD chromosomes, which disproportionately harbor genes with no clear *Fusarium* orthologues, leading to the hypothesis that they were gained through unknown horizontal gene transfer (HGT) events [44]. While asexual transfer of CD chromosomes has not been demonstrated in the FSSC, it has been shown experimentally in *F. oxysporum *[82].

## Conclusion

An extensive body of literature has connected many *Fusarium* infections, particularly ones suspected of being acquired nosocomially, to isolates resident in the patient indoor environment [21,38,41]. Similar to what has been observed in other common nosocomially acquired fungal pathogens, including *A. fumigatus *[83], we found no evidence differentiating clinical isolates from those collected from other sources, including biofilms in plumbing drains (Figure 2B). Although all known isolates of *F. keratoplasticum* are from highly anthropogenically influenced environments, nothing is known about a potential natural reservoir for this species [21,39,41]. While some STs show a broad geographic distribution, this MLST system may permit epidemiological inferences connecting patient and indoor environmental STs at a local scale. Diverse genotypes with patterns indicating historical recombination were observed, though there was also strong evidence for clonal expansion of epidemic STs. This mixed reproductive strategy, which is not unusual in fungal pathogens [28,73,84,85], enables the expansion of successful genotypes, at the same time allowing for adaptation to changing environments and antifungal treatment strategies. The detection of natural hybridization and introgression in this sample of isolates may be indicative of another prevalent mechanism permitting adaptation to the anthropogenic environments these fungi inhabit.

## Availability of supporting data

Nucleotide sequence data for *F. keratoplasticum* from the six new sequence markers are available through NCBI GenBank with the following accession numbers: locus 3968: JN585993-JN586184; locus 3972: JN586185-JN586372; locus 4081: JN586373-JN586556; locus 5437: JN586557-JN586711; locus 5439: JN586712-JN586899; locus 6512: JN586900-JN587087.

## Competing interests

The authors declare that they have no competing interests.

## Authors’ contributions

DPGS performed all fungal culturing, DNA extraction, marker design and validation, PCR, sequencing, alignment, and analyses and drafted the manuscript. DG and KO conceived of the study and participated in its design and coordination and helped to draft the manuscript. All authors read and approved the final manuscript.

## Supplementary Material

Additional file 1: Table S1 111 nine-locus sequence types and their frequencies based on the diversity of the 231 *F. keratoplasticum* isolates used in this study. Click here for file
