# Supplementary material for: Clonality, recombination, and hybridization in the plumbing-inhabiting human pathogen Fusarium keratoplasticum inferred from multilocus sequence typing
Source: BMC Evol Biol. 2014 Apr 26;14:91. doi: 10.1186/1471-2148-14-91 (PMC4026394; doi:10.1186/1471-2148-14-91)
Supplement: Additional file 1: Table S1 — 111 nine-locus sequence types and their frequencies based on the diversity of the 231 F. keratoplasticum isolates used in this study. [file 1471-2148-14-91-S1.docx]

Additional file 1: Table S1. 111 nine-locus sequence types and their frequencies based on the diversity of the 231 *F. keratoplasticum* isolates used in this study.

| **9-digit ST** | **Digitized nine-locus ST**^1^ | **No. Isolates** |
| --- | --- | --- |
| 2-a1 | 1-1-1-1-1-1-1-1-1 | 3 |
| 2-a2 | 1-1-1-1-1-1-1-2-1 | 1 |
| 2-b1 | 20-1-1-1-1-1-1-1-1 | 2 |
| 2-b2 | 20-1-1-1-2-6-1-1-6 | 2 |
| 2-c1 | 16-1-1-4-6-2-3-4-13 | 2 |
| 2-d1 | 20-1-4-1-2-2-1-1-13 | 23 |
| 2-d10 | 20-1-4-1-2-2-5-1-13 | 1 |
| 2-d11 | 20-1-4-1-1-2-1-1-10 | 1 |
| 2-d12 | 20-1-4-1-1-2-1-1-6 | 2 |
| 2-d13 | 20-1-4-1-2-2-1-1-6 | 1 |
| 2-d14 | 20-1-4-1-2-6-1-1-6 | 1 |
| 2-d16 | 20-1-4-1-2-18-1-1-1 | 1 |
| 2-d17 | 20-1-4-1-2-18-1-1-2 | 2 |
| 2-d18 | 20-1-4-1-13-2-1-3-2 | 1 |
| 2-d15 | 20-1-4-1-2-2-1-1-1 | 1 |
| 2-d2 | 20-1-4-1-2-18-1-1-13 | 49 |
| 2-d3 | 20-1-4-1-2-18-1-7-13 | 2 |
| 2-d4 | 20-1-4-1-2-2-1-7-13 | 2 |
| 2-d5 | 20-1-4-1-2-2-6-7-10 | 8 |
| 2-d6 | 20-1-4-1-2-8-6-7-10 | 1 |
| 2-d7 | 20-1-4-1-2-8-6-1-10 | 1 |
| 2-d8 | 20-1-4-1-2-2-6-1-10 | 9 |
| 2-d9 | 20-1-4-5-2-17-1-3-13 | 1 |
| 2-e1 | 18-1-1-1-1-1-1-1-6 | 1 |
| 2-f1 | 18-1-4-1-2-7-1-8-6 | 1 |
| 2-f2 | 18-1-4-1-2-7-1-1-6 | 4 |
| 2-f3 | 18-1-4-1-2-6-1-1-6 | 1 |
| 2-f4 | 18-1-4-1-6-6-1-8-6 | 1 |
| 2-f5 | 18-1-4-1-2-2-1-8-6 | 1 |
| 2-f6 | 18-1-4-1-2-2-1-1-13 | 6 |
| 2-f7 | 18-1-4-1-2-7-1-1-21 | 2 |
| 2-g1 | 14-1-4-3-2-2-1-1-8 | 1 |
| 2-g2 | 14-1-4-3-2-2-1-1-15 | 1 |
| 2-g3 | 14-1-4-5-2-7-1-1-5 | 1 |
| 2-g4 | 14-1-4-3-2-2-1-1-1 | 1 |
| 2-g5 | 14-1-4-3-2-16-8-1-5 | 2 |
| 2-h1 | 20-3-4-1-2-18-1-1-13 | 1 |
| 2-h2 | 20-3-4-1-2-2-6-1-2 | 1 |
| 2-h3 | 20-3-4-1-2-9-6-1-2 | 1 |
| 2-h4 | 20-3-4-1-2-2-1-1-2 | 1 |
| 2-h5 | 20-3-4-1-2-2-1-1-1 | 1 |
| 2-i1 | 22-1-4-1-2-6-1-1-11 | 1 |
| 2-i2 | 22-1-4-1-2-6-1-8-6 | 1 |
| 2-i3 | 22-1-4-1-2-2-1-1-6 | 1 |
| 2-i4 | 22-1-4-1-2-7-1-1-1 | 1 |
| 2-j1 | 4-1-6-3-2-2-1-1-3 | 1 |
| 2-k1 | 15-1-4-1-2-2-1-3-13 | 1 |
| 2-k10 | 15-1-4-8-2-8-1-3-2 | 1 |
| 2-k11 | 15-1-4-9-2-2-1-9-1 | 1 |
| 2-k12 | 15-1-4-1-2-2-1-3-2 | 2 |
| 2-k2 | 15-1-4-11-11-2-7-3-9 | 1 |
| 2-k3 | 15-1-4-10-11-2-7-3-16 | 1 |
| 2-k4 | 15-1-4-1-10-2-1-3-2 | 4 |
| 2-k5 | 15-1-4-1-10-2-12-9-2 | 3 |
| 2-k6 | 15-1-4-1-10-8-12-9-2 | 2 |
| 2-k7 | 15-1-4-1-10-2-1-1-2 | 1 |
| 2-k8 | 15-1-4-8-10-2-12-3-2 | 1 |
| 2-k9 | 15-1-4-8-10-2-1-1-2 | 1 |
| 2-l1 | 9-1-4-3-2-3-10-1-2 | 1 |
| 2-m1 | 10-1-1-6-2-4-10-10-10 | 1 |
| 2-n1 | 3-2-1-1-2-6-1-1-18 | 1 |
| 2-o1 | 14-2-4-3-2-2-8-1-3 | 2 |
| 2-o2 | 14-2-4-3-2-2-1-1-1 | 2 |
| 2-o3 | 14-2-4-3-2-7-1-1-1 | 1 |
| 2-o4 | 14-2-4-9-2-13-8-1-5 | 1 |
| 2-o5 | 14-2-4-12-8-7-1-1-1 | 1 |
| 2-p1 | 7-5-5-1-12-14-11-5-19 | 1 |
| 2-q1 | 5-5-10-12-2-11-1-1-4 | 1 |
| 2-r1 | 5-5-9-5-2-11-8-1-17 | 1 |
| 2-s1 | 8-1-4-3-9-3-8-1-5 | 1 |
| 2-s2 | 8-1-4-5-2-2-8-1-2 | 1 |
| 2-s3 | 8-1-4-12-2-2-8-6-2 | 1 |
| 2-s4 | 8-1-4-12-2-7-1-1-2 | 1 |
| 2-s5 | 8-1-4-3-2-16-1-1-8 | 1 |
| 2-t1 | 1-1-4-12-2-17-8-1-3 | 1 |
| 2-t2 | 1-1-4-1-2-2-1-1-2 | 1 |
| 2-t3 | 1-1-4-9-9-10-8-3-3 | 1 |
| 2-u1 | 5-3-9-3-5-7-1-1-1 | 1 |
| 2-v1 | 22-1-10-1-2-6-1-1-17 | 2 |
| 2-w1 | 7-1-5-2-6-2-1-1-14 | 1 |
| 2-x1 | 12-1-4-1-2-15-1-1-12 | 1 |
| 2-x2 | 12-1-4-1-6-5-1-1-2 | 1 |
| 2-aa1 | 21-1-4-1-3-2-1-1-2 | 1 |
| 2-cc1 | 6-4-4-7-7-6-4-3-7 | 1 |
| 2-ii1 | 14-1-11-3-2-7-8-1-13 | 1 |
| 2-ii2 | 14-1-11-3-2-10-8-4-17 | 1 |
| 2-ii3 | 14-1-11-3-2-7-8-1-1 | 1 |
| 2-ii4 | 14-1-11-3-2-7-1-1-5 | 1 |
| 2-jj2 | 14-1-6-5-2-6-8-1-1 | 1 |
| 2-kk1 | 13-2-4-3-2-16-9-1-5 | 2 |
| 2-ll1 | 11-2-4-3-2-7-1-1-8 | 1 |
| 2-mm | 14-6-4-5-2-7-1-1-5 | 1 |
| 2-nn1 | 11-6-4-3-2-7-1-1-2 | 1 |
| 2-oo1 | 2-1-4-1-2-6-1-1-22 | 1 |
| 2-pp1 | 8-1-11-9-2-10-10-1-13 | 1 |
| 2-pp2 | 8-1-11-3-2-7-8-1-3 | 1 |
| 2-pp3 | 8-1-11-9-2-7-1-3-3 | 2 |
| 2-qq1 | 1-1-11-12-2-7-1-1-1 | 1 |
| 2-qq2 | 1-1-11-3-2-7-1-3-3 | 1 |
| 2-rr1 | 4-2-4-9-2-16-1-1-2 | 1 |
| 2-ss1 | 7-3-3-9-2-12-1-1-15 | 1 |
| 2-tt1 | 8-1-2-3-2-17-1-1-2 | 1 |
| 2-uu1 | 5-1-4-3-2-7-8-1-1 | 3 |
| 2-vv1 | 15-1-7-1-10-2-12-3-2 | 1 |
| 2-vv2 | 15-1-7-1-10-2-1-3-2 | 3 |
| 2-ww1 | 22-1-5-1-2-2-1-1-6 | 1 |
| 2-xx1 | 14-1-11-3-2-6-1-1-2 | 1 |
| 2-yy1 | 1-1-5-1-2-2-2-1-23 | 1 |
| 2-aaa1 | 1-2-8-3-4-3-1-1-3 | 1 |
| 2-bbb1 | 17-1-4-1-2-2-1-1-13 | 1 |
| 2-ccc1 | 14-1-1-12-2-10-1-1-2 | 1 |

1. Order of the 9 loci: *TEF*-rDNA-*RPB2*-3968-3972-4081-6512-5439-5437. SNPs, microsatellites, and indel polymorphisms are included in allele assignments.
